# Contrasting new and available reference genomes to highlight uncertainties in assemblies and areas for future improvement: an example with monodontid species

**DOI:** 10.1186/s12864-023-09779-3

**Published:** 2023-11-20

**Authors:** Trevor T. Bringloe, Geneviève J. Parent

**Affiliations:** Laboratory of Genomics, Maurice Lamontagne Institute, Fisheries and Oceans Canada, Mont-Joli, QC Canada

**Keywords:** Cetacea, Narwhal, Beluga, *Monodon monoceros*, *Delphinapterus leucas*, Genomics, Long read sequencing

## Abstract

**Background:**

Reference genomes provide a foundational framework for evolutionary investigations, ecological analysis, and conservation science, yet uncertainties in the assembly of reference genomes are difficult to assess, and by extension rarely quantified. Reference genomes for monodontid cetaceans span a wide spectrum of data types and analytical approaches, providing the context to derive broader insights related to discrepancies and regions of uncertainty in reference genome assembly. We generated three beluga (*Delphinapterus leucas*) and one narwhal (*Monodon monoceros*) reference genomes and contrasted these with published chromosomal scale assemblies for each species to quantify discrepancies associated with genome assemblies.

**Results:**

The new reference genomes achieved chromosomal scale assembly using a combination of PacBio long reads, Illumina short reads, and Hi-C scaffolding data. For beluga, we identified discrepancies in the order and orientation of contigs in 2.2–3.7% of the total genome depending on the pairwise comparison of references. In addition, unsupported higher order scaffolding was identified in published reference genomes. In contrast, we estimated 8.2% of the compared narwhal genomes featured discrepancies, with inversions being notably abundant (5.3%). Discrepancies were linked to repetitive elements in both species.

**Conclusions:**

We provide several new reference genomes for beluga (*Delphinapterus leucas*), while highlighting potential avenues for improvements. In particular, additional layers of data providing information on ultra-long genomic distances are needed to resolve persistent errors in reference genome construction. The comparative analyses of monodontid reference genomes suggested that the three new reference genomes for beluga are more accurate compared to the currently published reference genome, but that the new narwhal genome is less accurate than one published. We also present a conceptual summary for improving the accuracy of reference genomes with relevance to end-user needs and how they relate to levels of assembly quality and uncertainty.

**Supplementary Information:**

The online version contains supplementary material available at 10.1186/s12864-023-09779-3.

## Background

Reference genomes provide a fundamental framework for evolutionary investigations (e.g. [1]), population level inferences (e.g. [2, 3]), hybridizations (e.g. [4]), and conservation and restoration science [5]. International consortiums now seek to generate complete and error-free reference genomes for all vertebrate species [6], an effort mirrored in other eukaryotic lineages such as fungi [7], algae [8], and arthropods [9]. Nearly two thirds of NCBI’s 28 k currently available eukaryotic genomes were released since the beginning of 2020 (data accessed March 2, 2023; https://www.ncbi.nlm.nih.gov/genome/browse/#!/overview/). Of these eukaryotic genomes, only 1% are listed as complete (all chromosomes present with no gaps), and only another 15% represent chromosomal level scaffolds. Of the complete genomes, only two represent animals i.e., human and *C. elegans*.

As reference genomes continue to accumulate, multiple assemblies for the same species are also becoming available. For example, the NOAA’s Cetacean Genome Project has amalgamated cetacean genomes from various resources, with most species featuring 2–4, and upwards of 8, available reference genomes (https://www.fisheries.noaa.gov/resource/data/cetacean-genomes-status). Depending on end-user needs, this can warrant careful consideration as to which reference genome ought to be leveraged for analysis. Completeness and contiguity are typically the basic properties used to assess reference genome quality. The completeness of a reference genome may be proxied by comparing total assembly length with the expected genome size, or through tallying expected single copy orthologues (e.g. BUSCO; [10]), and a movement towards more integrative quality metrics is also gaining traction [6, 11]. The contiguity of assembled sequences is commonly proxied by assembly statistics such as number of contigs/scaffolds, N50, L50, and % gaps if a reference is scaffolded (for definitions, among other relevant ones, see Table 1). Thus, a more contiguous reference genome has a lower number of contigs, gaps, and L50 value, and a higher N50 value. Metrics may be inflated, however, if sequence data is incorrectly oriented and joined. These mis-joins can be corrected to some degree with downstream datatypes and methods (e.g. Hi-C and the 3D-dna workflow; [12, 13]), but because novel reference genomes are typically sought after and constructed for a single individual, mis-join errors remain difficult to detect without a meaningful point of comparison. The comparison of multiple reference genomes in target species could help pinpoint discrepancies as potential errors. This would add another dimension to estimating reference genome quality by highlighting regions of uncertainty in reference genomes.

**Table 1:** Definitions for terms used in the current study

Term	Definition
**Data types**	
Short reads	Accurate sequences of DNA typically 150 bp in length and typically generated on Illumina platforms [14]. Sequences may be paired or unpaired depending on whether both ends of DNA fragments were sequenced
Linked reads	Short reads, but with molecular barcodes that tag reads from the same DNA fragment, creating “read clouds” that leverage long range information [15]. Sometimes referred to as 10 × Linked reads after 10 × Genomics, a company that provided this type of sequencing prior to 2020
Long reads	Sequences produced directly from long fragments of DNA, thus providing long range information in the form of intact reads. Typically generated using PacBio or NanoPore platforms, long reads are historically more error prone compared to short reads, though read accuracy continues to improve (e.g. PacBio HiFi reads; [14])
Chromosome conformation capture	A method used to map spatial organization of chromatin across genomes [16]. A suite of techniques can be used to cross link loci and sequence DNA fragments as paired-end short reads linked by unknown proximity. The higher order structure of sequences (e.g. chromosomes) can be inferred because loci interactions increase with linear proximity on the genome. Data is generated on similar platforms to short read sequences, e.g. Illumina
Optical genome mapping	A restriction enzyme is applied to highly intact DNA and the lengths and order of fragments are measured. This information is used to guide the order and orientation of assembly fragments by matching patterns in the occurrence of sequence motifs [17, 18]. Note, the data represents mapping information or physical locations of sequence motifs, not sequence data. Bionano is currently the main provider for optical genome mapping services
**Reference genome quality**	
K-mer	Substrings of length k within DNA sequence data
Coverage	The number of times, on average, a genomic region or complete genome has been sequenced. Oftentimes synonymous with the depth, or number, of uniquely overlapping reads in a dataset
Contig	A DNA sequence assembled by overlapping k-mers or reads
Scaffold	Contigs ordered and oriented into longer sequences, typically with gaps represented as Ns in between contigs [19]
Contiguity	The level to which a reference genome is assembled into continuous sequences representing DNA, a genome fragmented into a larger amount of smaller sequences being less contiguous
*Quantitative parameters*	
N50	The minimum sequence length above which 50% of the reference genome is represented. A proxy for contiguity
L50	The minimum number of sequences within which 50% of the reference genome is represented. A proxy for contiguity
Completeness	The proportion of the genomic sequences captured in a reference assembly. This is typically benchmarked using the proportion of observed vs expected single copy orthologues appearing in an assembly (i.e. BUSCO scores; [10])
*Qualitative parameters*	
Accuracy	A general term to scale the match between an assembly and a hypothetical complete and error-free assembly
Precision	A general term to scale the replicability of the assembly using similar or alternative methods
Certainty/uncertainty	A general term to scale the confidence surrounding a genomic sequence or assembly
Error/mis-join/mis-assembly	An incorrect inference regarding the order and/or orientation of a particular genomic sequence
Discrepancy	An inconsistency between two reference genomes which could be due to an error or inter- or intraspecific variation
**Discrepancies**	
Debris	Segments of DNA, typically contigs, not assimilated into higher order scaffolding of chromosome sequences
Gaps	Runs of Ns, typically 10-100, that appear between contigs within scaffolds, representing uncertainty between the adjoining sequences
Translocation	A unique DNA segment appearing on different chromosomes between two assemblies
Inversion	A unique DNA segment running in opposite directions between two assemblies
Relocation	Unique DNA segments appearing in a different order between two assemblies
**General terms**	
Restriction enzyme	A protein that cleaves DNA at sites with a particular sequence, or restriction site
Orthologous	A DNA segment or gene appearing in separate species and inherited from a common ancestor, typically retaining similar function
Repetitive element	Patterns of DNA sequences that occur as multiple copies throughout a genome
Transposable element	DNA sequences, typically genes, that can move location within a genome
Reference genome	A representation or estimation of the entire genomic sequence of a species or individual
End-user	Someone seeking to leverage a previously generated reference genome for applied purposes. For example, an end-user might use a reference genome to map sequences and call variant positions in a set of samples

Data types, bioinformatic workflows, and biological features of target organisms (e.g., repetitive elements) all drive disparity in the quality of reference genomes, which end-users must consider. For instance, it is widely accepted that highly contiguous assemblies require long distance information that can bridge repetitive elements [6]. Examples of long distance information commonly used are linked reads, i.e. short read clouds allowing the resolution of longer DNA segments, or Nanopore or PacBio long reads which are direct, but may be prone to error (Table 1). Assembled reads, or contigs, are also typically ordered and oriented into longer DNA sequences called scaffolds. Again, different data types and approaches can drive disparity in reference genome quality. Chromosome conformation capture represents a suit of approaches that can infer the chromosomal scale structure of DNA sequences [20], while optical genome mapping provides ultra-long range conformation information by mapping restriction sites or sequence motifs along high molecular weight DNA and applying this map to scaffold contigs [17, 18]. The number of bioinformatic tools available to achieve these steps are numerous (reviewed by [20, 21]), creating multiple pathways towards constructing a reference genome, along with different end-points that approximate the true genomic sequence of an organism.

Several reference genomes are available for monodontids, a family of cetaceans comprising two genera/species, the beluga whale (*Delphinapterus leucas*) and narwhal (*Monodon monoceros*). These resources derive from multiple research groups depositing data on NCBI [1, 2, 22] or the organization DNAzoo (https://www.dnazoo.org/). Reference genomes for monodontids span a large spectrum of contiguity and data types used, making beluga and narwhal ideal species from which to derive broader insights related to discrepancies and regions of uncertainty in reference genome assembly. And while poor to moderate genomic representations may serve some biological investigations such as variant calling for population genomics, highly accurate and contiguous genomes are desirable to open new opportunities for evolutionary investigations (e.g. [1]).

Belugas are circumpolar in their distribution, with isolated populations extending as far south as the St. Lawrence Estuary and Gulf in the Northwest Atlantic. It as a nutritional and culturally significant resource for northern indigenous communities [23], and is of priority status for conservation under increasing climate change related threats [24–27]. Two reference genomes are available for this species. Jones et al. [22] were the first to forward a reference genome for beluga using 10 × linked short reads. In order to improve on the assembly, Jones et al. [22] used alignment and k-mer based iterative scaffolding and gap filling guided by sequences produced using short read assemblies of the target and daughter individuals. The initial 2017 reference assembly has since undergone several iterations, with the L50 now at 31.183 Mb (Table 2), though details regarding the methods underlying these improvements are, to the best of our knowledge, not available. DNAzoo also provided a reference genome using Hi-C data and the 3D-dna workflow [12, 13] to scaffold the Jones et al. [22] v2 assembly into chromosomal length sequences (Table 2; https://www.dnazoo.org/assemblies/Delphinapterus_leucas). This reference genome along with methods associated with the assembly (including Jones et al. [22] v2 improvements), though broadly available through DNAzoo (https://www.dnazoo.org/methods), remain unpublished, presenting barriers for end users to specifically understand the context surrounding how this reference genome was constructed.

**Table 2:** Assembly parameters for *Delphinapterus leucas* (beluga) and *Monodon monoceros* (narwhal)

Species	Reference (accession)	Name	Sequences	Assembler	Scaffolding approach
*Delphinapterus leucas*	Jones et al. [22] (ASM228892v3)	Dl_Jones_	10 × linked and illumina short reads	ABYSS, Supernova	Iterative scaffolding with RAILS, LINKS
	DNA zoo (ASM228892v2_HiC)	Dl_zoo_	Jones et al. (2017) v2 assembly, Hi-C	ABYSS, Supernova	Juicer, 3D-dna workflows
	S_20_00693 (GCA_029941415)	Dl_3_	PacBio CLR (36x), illumina short reads (92x), Hi-C (26x)	Flye v.2.9	Juicer, 3D dna workflows
	S_20_00702 (GCA_029941435)	Dl_4_	PacBio CLR (40x), illumina short reads (86x), Hi-C (26x)	Flye v.2.9	Juicer, 3D dna workflows
	S_20_00703 (GCA_029941455)	Dl_5_	PacBio CLR (53x), illumina short reads (75x), Hi-C (26x)	Flye v.2.9	Juicer, 3D dna workflows
*Monodon monoceros*	Westbury et al. [2] (GCA_005125345.1)	Mm_West_	Illumina short reads, cross species mate pairs	SOAPdenovo v.2	Cross-species scaffolding using Jones et al. [22]
	Damas et al. [1] GCA_005190385.3	Mm_Damas_	PacBio CLR (40x), Dovetail Omni-C reads, Bionano	FALCON-Unzip	Dovetail proprietary HiRise workflow, Bionano
	0422/S_20_00708 (GCA_029941395)	Mm_3_	PacBio CLR (58x), Illumina short reads (40x), Hi-C (39x)	Flye v.2.9	Juicer, 3D dna workflows

Narwhal is the closest living relative to the beluga, and though they are from separate genera, a hybrid individual was confirmed from morphological (i.e. skull remains; [28]) and molecular data [4]. Narwhal is listed as a species of special concern by the Committee on the Status of Endangered Wildlife in Canada [29], and already depleted levels of diversity coupled with contracting habitat under climate change continue to threaten this species [30]. In order to construct the first reference genome for narwhal, Westbury et al. [2] used cross-species mate paired libraries as long-range information, which were guided using the beluga reference genome of Jones et al. ([22]; Table 1). As such, scaffolding errors in the Jones et al. [22] assembly may have carried through into the reference genome of narwhal. More recently, Damas et al. [1] presented a notably improved reference genome for narwhal, assembled using PacBio, Omni-C, and Bionano data (Damas pers. comms), with chromosomes scaffolded from only 413 contigs (see Table 1 for definitions). Such a laudable effort could serve as a model for improving the reference genome of beluga, but details are scant on the exact methods used to assemble and scaffold the data, and much of the bioinformatic workflows sit behind proprietary programs such as Dovetail’s HiRise software (the open-source version has not been maintained since 2015 [20]). A critical assessment of the assembly methods used across monodontid reference genomes is therefore challenging for end-users unfamiliar with assembly methods.

In this study, we provide four new reference genomes for monodontidae and compare these new and past resources to quantify discrepancies between assemblies. In order to meet this aim, we assembled genomes for beluga (*n* = 3) and narwhal (*n* = 1) using PacBio Continuous Long Reads, which were polished (i.e. base call corrected) using Illumina paired-end short reads, and scaffolded using Hi-C data. We then compared our new and published reference assemblies (Jones et al. [22], DNAzoo, Westbury et al. [2], Damas et al. [1]) with respect to completeness, contiguity, data types, assembly workflow, and discrepancies. By quantifying these discrepancies in monodontid reference genomes, we were able to derive broader insights related to the challenges of constructing novel reference genomes and the potential errors users must account for depending on their research question and analytical needs.

## Results

### Four new reference genomes for monodontidae

#### Beluga

**Fig. 1:**
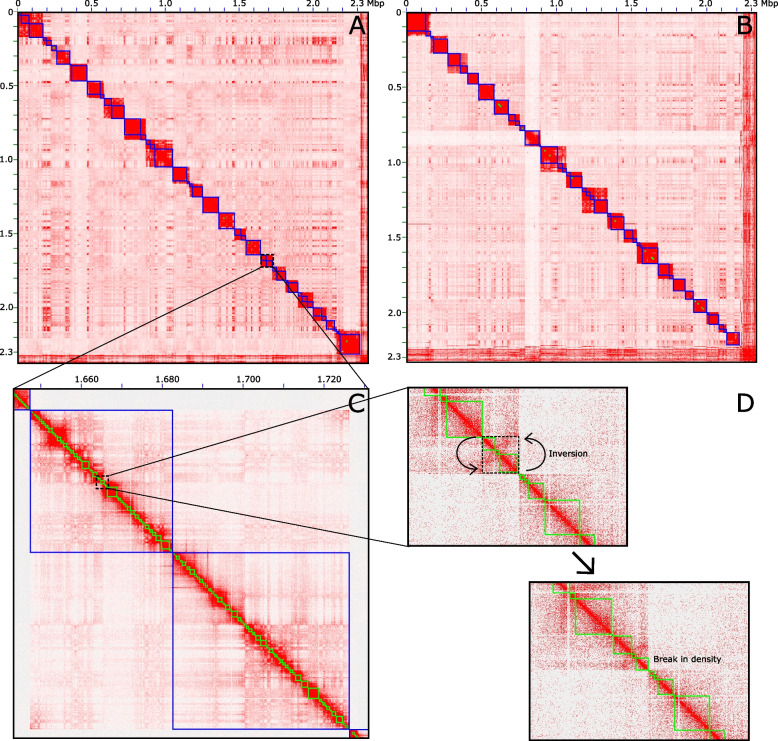
Hi-C contact matrices for (**A**) *Delphinapterus leucas* (beluga) and (**B**) *Monodon monoceros* (narwhal) PacBio assemblies. The blue boxes delineate super scaffolds, which are assigned to chromosomes based on contact densities. **C** Most chromosomes must be broken into several super scaffolds due to breaks in densities and ambiguous ordering of large domains. **D** Localized misassemblies also require rigorous and comprehensive manual intervention. Here, a block of inverted contigs requires correction. Moreover, a break in contact densities suggests there are missing sequences (likely allocated to debris) that ought to be incorporated; regional contact densities may nonetheless support scaffolding at this junction

We generated three new beluga reference genomes (Dl_3_, Dl_4_, Dl_5_) with genome sizes varying between 2,379 and 2,404 Mbp and slightly greater than Dl_Jones_ and Dl_zoo_ (Table 3). We obtained 21 autosomal chromosomes and the X chromosome for the three new reference genomes (Fig. 1). Chromosomal scale structure was evident in the Hi-C contact matrices, but breaks in contact densities were also present (Fig. 1A, B, C). Ambiguous large-scale ordering was common, oftentimes making up two large segments of a given chromosome (Fig. 1C). The new reference genomes were ~ 94.6% complete based on BUSCO scores with > 88.6% single copy BUSCOs predicted. BUSCO scores were higher by ~ 3.75% in the Dl_Jones_ and Dl_zoo_ assemblies. We predicted 42.2% of the new reference genomes represent repeat elements, and predicted 19,545–19,734 genes with 93.5% of AED scores ≤ 0.5.

**Table 3:** Assembly statistics for *Delphinapterus leucas* (beluga) and *Monodon monoceros* (narwhal). For novel reference genomes presented here, the contig statistics represent initial metrics following long read assembly using Flye. For BUSCO scores based on the vertebrata database (*n* = 3,354 BUSCOs), S = single, D = duplicated, F = fragmented, M = missing. Note, BUSCO for previously published assemblies were reanalysed here to ensure consistency

Species	Names	Total length (Mbp)	Contig/scaffold total number	Gaps (%)	Max contig/scaffold length (Mbp)	Contig/scaffold L50	Contig/scaffold N50 (Mbp)	BUSCO score (S:D:F:M)
*Delphinapterus leucas*	Dl_Jones_	2,363	29,098/5,906	1.518	1.711/120.97	3,611/21	0.197/31.183	92.5:2.5:2.0:3.0
	Dl_zoo_	2,357	35,102/6,972	1.264	1.083/182.318	4,473/9	0.159/107.97	92.4:2.5:2.0:3.1
	Dl_3_	2,404	20,104/9,110	0.056	2.386/135.447	2,190/12	0.316/88.022	88.6:2.4:3.6:5.4
	Dl_4_	2,397	12,979/5,851	0.038	3.224/135.399	1,249/12	0.551/87.682	88.6:2.8:3.2:5.4
	Dl_5_	2,379	10,046/4,370	0.032	5.618/134.683	1,035/12	0.657/87.683	88.8:2.7:3.2:5.3
*Monodon monoceros*	Dl_West_	2,351	813,468/21,006	8.259	0.135/7.088	63,731/464	0.010/1.483	90.8:2.1:3.2:3.9
	Mm_Damas_	2,342	414/101	0.001	83.581/182.209	36/9	22.031/108.564	92.6:2.4:1.9:3.1
	Mm_3_	2,337	9,252/6,465	0.035	5.696/131.705	861/12	0.708/83.77	84.4:2.6:4.2:8.8

All contiguity parameters at the contig level showed improvements with the new reference genomes compared to Dl_Jones_ and Dl_zoo_ (Table 3). The new beluga genomes reduced the number of contigs compared to Dl_Jones_ and Dl_zoo_ by as much as 72% (Table 3), while the proportions of gaps were reduced from ~ 1.5% to < 0.06%. Maximum contig lengths increased by a factor of 1.39 to 5.19, L50s were reduced by factors of 0.59 to 4.32, and N50s increased by a factor of 1.60 to 4.13 for new reference genomes compared to Dl_Jones_ and Dl_zoo_ (Table 3).

Scaffolding metrics for the new reference genomes were greater or reduced compared to Dl_Jones_ and Dl_zoo_, respectively (Table 3). The total number of scaffolds was reduced by 37% for Dl_5_ compared to Dl_zoo_, but was increased by 35% in Dl_3_ compared to Dl_Jones_ (Table 3). The reference genomes Dl_3,4,5_ improved max scaffold length by a factor of 1.13 compared to DNA_Jones_, while Dl_zoo_ max scaffold length was greater by a factor of 1.35 compared to the new reference assemblies. The L50 of the Dl_zoo_ and Dl_Jones_ were also smaller (9) and greater (21) compared to the new reference genomes (12, Table 3). The new beluga reference genomes converged to a scaffold N50 of ~ 88 Mbp, an increased value by a factor of 2.80 compared to Dl_Jones_ (31 Mbp), but 19% smaller than the N50 of Dl_zoo_ (108 Mbp; Table 3).

#### Narwhal

We generated one narwhal reference genome (Mm_3_) with a genome size of 2,337 Mbp and slightly smaller than those from Mm_west_ and Mm_Damas_ (Table 3). As with beluga, we obtained 21 autosomal chromosomes and the X chromosome for this new reference genome (Fig. 1). The new reference genome has improved contiguity and scaffolding parameters compared to Mm_west_, but not compared to Mm_Damas_ (Table 3). The Mm_3_ reference genome was 91.2% complete based on BUSCO scores (84.4% single copy), which was notably less compared to Mm_west_ and Mm_Damas_, by as much as 8.2%. We predicted 42.5% of Mm_3_ to represent repeat elements, a nearly identical value as Mm_Damas_ (which we predicted at 42.3%). We also predicted 21,086 genes for Mm_3_ with 97% of gene models with AED scores ≤ 0.5.

The new reference genome for narwhal similarly differed greatly in contiguity with the published reference genomes. The Mm_3_ reference genome reduced the number of contigs and scaffolds reported for Mm_west_ by 98.8% and 69.2%, respectively. Contiguity statistics showed similar levels of improvement for contrast between Mm_3_ and Mm_west_: 1) max contig and scaffold lengths were increased by factors of 42 and 19, respectively, 2) contig N50 and L50 were improved by factors of 71 and 74, respectively, and 3) scaffold N50 and L50 improved by factors of 56 and 39, respectively. In contrast, the Mm_Damas_ reference genome reduced the number of contigs and scaffolds compared to Mm_3_ by 95.5% and 98.4%, respectively. Comparing Mm_Damas_ to Mm_3_, max contig and scaffold lengths increased by a factors of 15 and 1.35, respectively, contig N50 and L50 increased and decreased, respectively, by factors of 31 and 24 and scaffold N50 and L50 increased and decreased, respectively, by a factor of 1.3 (Table 3).

### Comparisons of monodontid reference genomes

**Fig. 2:**
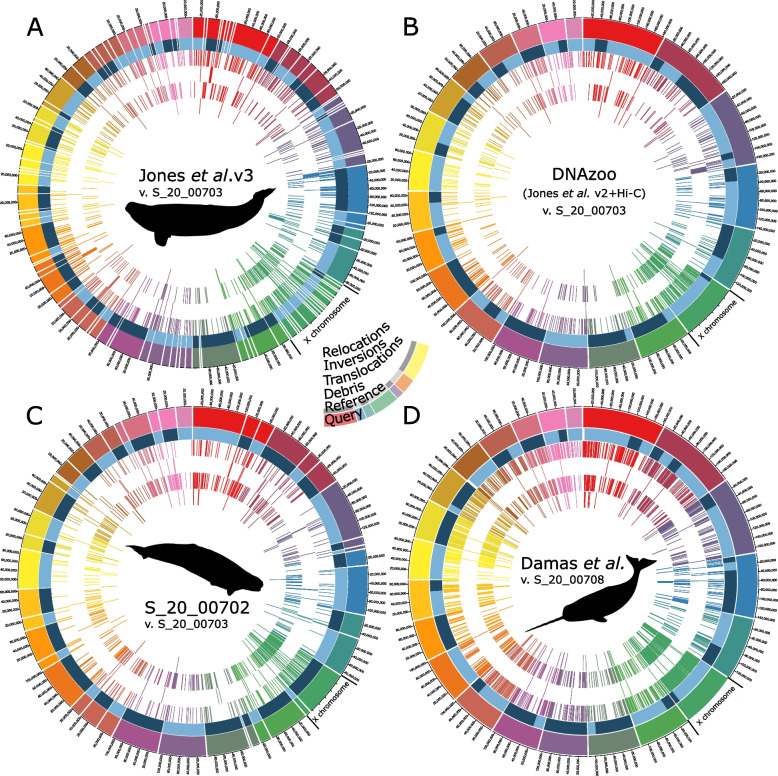
Discrepancies amongst reference genomes for (**A**-**C**) *Delphinapterus leucas* (beluga) and (**D**) *Monodon monoceros* (narwhal). The light and dark blue inside ring depicts the reference scaffolding structure (Dl_5_ for beluga, Mm_3_ for narwhal); alternating light and dark indicates a break in the super scaffolds making up chromosomes. Here, debris depicts small segments of DNA assembled in the query, but not in the reference. Translocations refer to segments of the query mapping to different reference chromosomes. Inversions refer to segments of the query mapping in a direction opposite to the dominant mapping direction for a given reference super scaffold. Relocations refer to segments of the query that appear out of order compared to the reference (see Table 1 for more definitions)

Our second aim was to quantify discrepancies between new and published reference genomes from both species. Discrepancies in the forms of debris, translocations, inversions, and relocations (Table 1) were present, and at times abundant, across all pairs of genomes evaluated (Table 4, Fig. 2). Several trends were notable for each discrepancy type. First, discrepancy in the form of small segments of DNA incorporated in one reference, but not the other (debris) was consistent across the beluga reference genomes, accounting for 0.3–0.6% of the total assemblies (Table 4, Fig. 2). This percentage was considerably higher in the comparison of Mm_3_ to Mm_Damas_; 2.5% of Mm_Damas_ was represented as smaller contigs/scaffolds relegated to debris in the new narwhal reference genome (Table 4, Fig. 2D). Relocations accounted for a small percentage of the discrepancies detected across beluga reference genomes scaffolded with Hi-C data (0.1% of total references). This proportion was slightly higher in the comparison between Dl_5_ and Dl_Jones_ (0.8%; Table 4, Fig. 2A), while the comparison of Mm_3_ and Mm_Damas_ was in between these values (0.35%). Translocations were diminished when comparing Dl_Jones_ and Dl_zoo_ to the Dl_3,4,5_ assemblies, but nonetheless accounted for a relatively small percentage of discrepancy (1.35%, reduced to 0.1% of total assembly; Table 4, Fig. 2A, B). Translocations were similarly low in the comparison of Mm_3_ and Mm_Damas_ (0.05% of total assemblies). Inversions appeared to account for the bulk of discrepancy across all reference comparisons. In beluga, inversions accounted for 1.0–1.8% of the total length of references (Table 4, Fig. 2A,B,C), and appeared to be highest when comparing Dl_3,4_ to Dl_5_. In narwhal, this proportion was greater in the comparison of Mm_3_ and Mm_Damas_, accounting for 5.3% of the total assemblies.

**Table 4:** Comparison of discrepancies between beluga (*Delphinapterus leucas*) and narwhal (*Monodon monoceros*) reference genomes. For beluga, assemblies were compared to Dl_5_, as this was the most contiguous; for narwhal, the assembly of Damas et al*.* [1] was compared to Mm_3_. Discrepancies as a percentage of the total evaluated reference genome are presented in parentheses

Assembly	Debris	Translocations	Inversions	Relocations	Unassessed	Congruent
*Delphinapterus leucas*						
Dl_Jones_	7,096,636^a^ (0.3)	31,779,574 (1.35)	29,465,224 (1.25)	18,044,276 (0.8)	109,570,873 (4.6)	2,166,825,960 (91.7)
Dl_zoo_	11,212,633 (0.5)	14,447,594 (0.6)	23,928,201 (1.0)	1,165,563 (0.1)	116,520,692 (4.9)	2,189,291,240(92.9)
Dl_3_	13,985,070 (0.6)	4,419,089 (0.1)	43,013,607 (1.8)	1,546,419 (0.1)	274,690,520(11.4)	2,068,226,002(86.0)
Dl_4_	10,800,991 (0.4)	1,403,102 (0.1)	40,843,164 (1.7)	1,899,502 (0.1)	191,862,255 (9.2)	2,122,477,807(88.5)
*Monodon monoceros*						
Mm_Damas_	59,103,779 (2.5)	909,121 (0.05)	124,283,977 (5.3)	7,848,688 (0.35)	115,575,344 (4.9)	2,034,233,699 (86.9)

^a^Only includes debris from reference Dl_5_ as Jones et al. [22] v3 was not assembled into chromosomes using Hi-C data

**Fig. 3:**
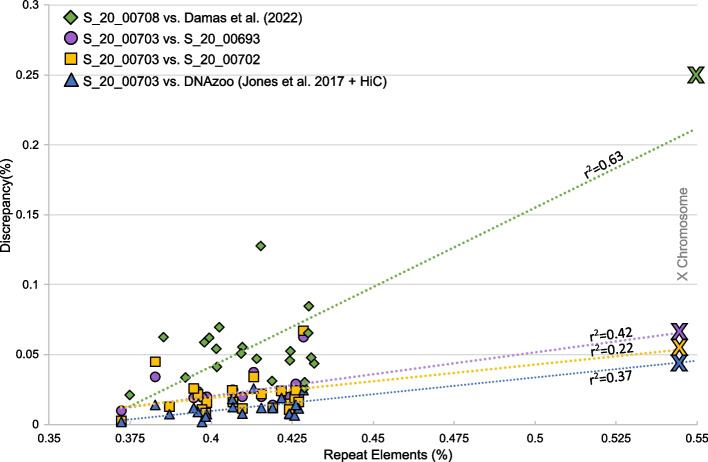
Regression analyses confirming a linear relationship between % repeat elements and % discrepancy across assemblies (*p* < 0.003 for each dataset, except for the comparison of Dl_4_ and Dl_5_ [*p* = 0.025]). Individual data points represent separate chromosomes. Points representing the X sex chromosome are depicted as Xs

Discrepancies also appeared to be non-randomly distributed within a given reference, and were disproportionately high for the X chromosome (Figs. 2 and 3). A significant linear relationship was generally confirmed between discrepancies and repetitive elements as a percentage of total base pairs at the chromosome level, with r^2^ values of 0.22–0.63 and *p*-values < 0.003, except for the comparison of Dl_4_ and Dl_5_ when correcting for multiple tests (*p* = 0.025; Fig. 3). The relationship was not significant when excluding the X chromosome.

## Discussion

**Fig. 4:**
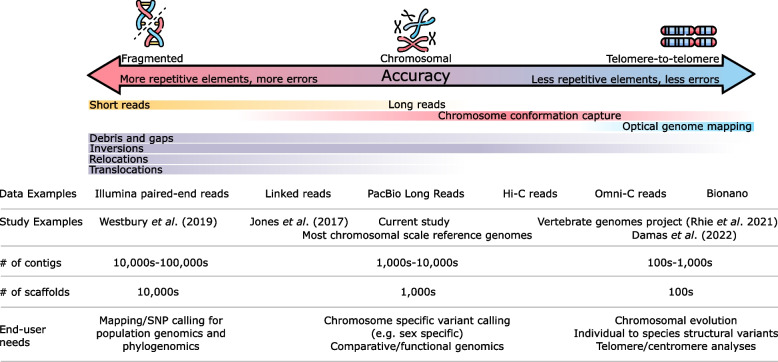
Hypothesized conceptual summary of reference genome accuracy, with specific reference to the construction of monodontid reference genomes. Note, in general, the percentage of repeat elements for a given genome skew accuracy in an inverse fashion. Errors are expected to diminish as more datatypes are combined, particularly those that resolve chromosomal scale ordering. We hypothesize debris is replaced, to a degree, by inversions, the latter of which feature a “long-tail” of persistence as datatypes are improved. End-user needs listed here may be carried forward with improved accuracy, but not backwards. Note, assembly accuracy approaches telomere-to-telomere assemblies, but fully resolving these regions remains the exception rather than the rule [6]. We also assume accuracy scales with the genetic distances proxied by the data types listed here, and acknowledge that assembly and scaffolding methods represent another variable in assembly accuracy not captured here. Images: Flaticon.com

A reference genome is an estimation subject to variation and errors introduced through various biological features, data types, and bioinformatic workflows [6, 31]. As novel reference genomes continue to become generated at an accelerating pace and leveraged by a wider user-base, it is also becoming increasingly imperative end-users understand associated limitations. In particular, assembly quality is typically proxied according to completeness and contiguity, but the context of uncertainty surrounding the order and orientation of sequences is rarely available for end-users. Here, we compared new and published reference genomes for beluga and narwhal and demonstrated putative plateaus to assembly accuracy which we link to different combinations of data types (Fig. 4). Long read assemblies in combination with chromosome conformation capture appear to correct scaffolded contigs with incorrect order and/or chromosomal assignment, but fall short of systematically correcting the orientation of contigs (i.e., inversions). The latter, along with systematically eliminating gaps, require added layers of long read information, for instance, in the form of optical genome mapping. Overall, our comparative analyses of reference genomes showed that the three new reference genomes for beluga are more accurate compared to published reference genomes on account of data types employed and extensive manual curation. We believe, however, that the new narwhal genome is less accurate than the reference genome from Damas et al. [1]. Our work serves as a checkpoint on the road to improving reference genomes for monodontids. We also provide considerations regarding drivers of uncertainties and avenues for their reduction in reference genomes. While our focal point here is monodontid reference genomes, we believe our discussion contributes to the overarching narrative surrounding the construction of improved reference genomes of non-model species.

### Aspects of reference genomes improved through long reads and chromosome conformation capture

Long reads improved monodontid assemblies compared to earlier short read assemblies in several ways. Contig level contiguity was improved by several orders of magnitude comparing Mm_West_ to Mm_3_, while these same parameters were improved several folds for beluga (Table 3). As expected, long reads bridge the junction between repetitive elements, which would otherwise result in broken assembly graphs constructed using short reads. The necessity for long reads was made obvious in the analyses of Rhie et al. [6], who demonstrated a 30–300 fold discrepancy in contig length when assembling Anna’s hummingbird from various read data types and assembly algorithms. Ou et al. [31] also demonstrated notable improvements in contiguity for an inbred line of maize when assembling from long read datasets with subread N50 lengths of 21 kbp and 40–75 × coverage compared to datasets with subread N50 of 11 kbp. The subread N50s of our study were approximately 14-17 kbp, suggesting an important avenue for improving the monodontid reference genomes presented here is to assemble read datasets with larger subread N50.

Hi-C data was also crucial towards properly assigning contigs to chromosomal level scaffolds (Fig. 1). Long range contact information in the form of Hi-C data or its derivatives have been indispensable for resolving chromosomal scale scaffolding in a wide range of eukaryotic taxa [16], ranging from other marine mammals such as sperm whale [3], to multicellular protists (e.g. kelp; [32]), to complex plant genomes (e.g. giant sequoia; [33]). The relative success of Hi-C analysis is contingent, in part, on the initial assembly of long contigs [21, 34], making long reads an indispensable foundation for high quality assemblies. The combination of long reads and Hi-C data also resolved several scaffolding errors present in Dl_Jones_ and Dl_zoo_, reducing the amount of translocated and relocated DNA segments by 89–96% (Table 3; Fig. 2). Note that these discrepancies represented a small number of errors that accounted for large segments of misplaced sequence data (figshare: https://doi.org/10.6084/m9.figshare.23227595.v1). While large segments (> 10 kbp) of misplaced DNA are putatively the result of assembly and/or scaffolding errors, some smaller segments of translocated and relocated sequences could be attributable to natural causes, such as transposable elements [35, 36].

We attempted to follow best practices summarized by Jung et al. [21, 34] in order to construct new monodontid reference genomes. Best practices are ideal by design, oftentimes at the expense of practicality. Practical considerations include budget, time, and access to high quality tissue for DNA extraction. Given these factors are limited for non-model organisms, certain assembly metric checkpoints simply may not be feasible; for instance, we were unable to procure a minimum contig N50 of 1 Mbp as recommended by Jung et al. [21] prior to scaffolding. In practice, many assemblies must move onto scaffolding despite less-than-ideal contig contiguity. Indeed, the central driving force behind the 3D-dna workflow is to produce chromosomal scale assemblies of eukaryotic organisms using a combination of highly affordable short reads and Hi-C data ([13]; https://www.dnazoo.org/). Given assembly quality is impacted by practical limitations in resources, end-users will encounter errors woven into the fabric of a given reference genome. Our results highlight this caveat, the relevance of which depends on end-user needs (Fig. 4).

### Breaks in Hi-C contacts reflect disparity between the realized and true contiguity

Our assemblies for beluga also improved on accuracy by breaking chromosomal level scaffolds where Hi-C contact densities are weak or ambiguous (Fig. 1A, C). Breaks in contact densities point to segments of DNA that failed to be incorporated into the scaffolding. This can be partially driven by contig lengths that do not meet the threshold criteria of the 3D-dna workflow (default is 15 kbp for inclusion). Some regions of DNA otherwise lacked contact information due to ambiguous mapping of reads to repetitive elements. Oftentimes, a break in contact densities cleaved chromosomes into two or more super scaffolds. The order and orientation of these large scaffolds were unclear either because contact densities were uniform across segments, or densities supported multiple configurations. Under circumstances where a particular configuration could not be supported over another by comparing intraspecific reference genomes, we took the conservative approach and broke chromosomes into several blocks (Fig. 2B). The current practice is to define scaffolding according to sharp drops in contact densities that delineate chromosomes, as was the case with DNAzoo’s scaffolding of the Jones et al. [22] v2 beluga assembly. We believe our reference genomes are closer to the true N50, and that the contiguity statistics of the DNAzoo reference are to some degree inflated because there is disparity between the realized and true N50 and L50 values. Ultimately, we were more conservative with our higher order scaffolding.

End-users should be wary of assembly errors backed by the drive for chromosomal scale scaffolds, which could be a widespread issue in reference genome construction. Chromosomal scale scaffolding of reference genomes generate a higher number of errors, as more connections invites more opportunity for mis-joins, making accuracy the currency for contiguity. Indeed, Rhie et al. [6] show that manual curation remains a crucial step towards improving reference genome accuracy, with 1000 s of interventions required across the 19 genomes they assessed. The presentation of novel genomes, for which chromosomal scale assembly is often expected and emphasized in publication titles, must take additional measures to ensure scaffolding practices do not potentially overreach, as for monodontid reference genomes (Fig. 2C, D). End-users must equally be cautious before reaching for the perceived “most contiguous” assembly to apply in their research. In the application of Hi-C data, the 3D-dna and Juicebox packages [12, 13, 37] are indispensable tools towards manually curating scaffolding errors introduced through equally necessary large-scale automation of bioinformatic workflows.

### Long reads and Hi-C data as a means, not an end, towards error-free reference genomes

Despite the improvements to accuracy discussed above, the new assemblies generated in our study still left much to be desired in the direction of error-free (or nearly error-free) representations of monodontid genomes. Debris, shorter sequences not incorporated into chromosomes, persisted in new assemblies and stood in stark contrast to the nearly gapless Mm_Damas_ (Table 2). Debris, to some degree, likely represented random variation across datasets in their ability to resolve repetitive elements, which would be driven by intraspecific variation both in sequence and the distribution of read lengths. In practice, this translates into repetitive elements being bridged in some assemblies and not others; the decline in the new beluga assembly contiguity with contig coverage was a telling sign ([31]; Table 1]. As such, debris were likely associated with repeat rich and difficult to resolve genomic regions such as telomeres, centromeres, and sex chromosomes ( [38]; X chromosome as demonstrated here; Figs. 2 and 3), genomic regions that would also disproportionately contain the other types of errors investigated here. Indeed, we found the relationship between the amount of discrepancy detected for a given pair of assemblies at the level of chromosomes was positively associated with the amount of repetitive elements (Fig. 3), though admittedly this relationship hinged on the notably higher proportion of repetitive elements of the X chromosome. The larger assembly size of Dl_3_ was also accounted for in debris, which constituted a larger proportion of repetitive elements compared to Dl_5_ (67 vs 54%, respectively).

Inversions also persisted in the new monodontid assemblies, accounting for 1.0–5.3% of the total length of reference genomes (Table 2). Many inversions were visually confirmed and corrected by inspecting the Hi-C contact maps (Fig. 2D). These corrections, however, only reduced the amount of inversions by 5.8–15.7 Mbp across the long read beluga and narwhal reference genomes assembled here. Interestingly, the distribution of inversion sizes peaked at ~ 20 kbp and were much less frequent at sizes 100 + kbp. When comparing beluga reference genomes, only 15–20% of inversions were greater than 100 kbp, but these accounted for approximately half of the total length of inversions. Consequently, infrequent large inversions nonetheless added substantially to the total amount of discrepancy. These results also suggest most inversions are concentrated in smaller contig sizes, close to the default 15 kbp threshold for inclusion in the 3D-dna workflow. Smaller contigs are relegated to debris as they are difficult to place and orient due to a lack of Hi-C contacts [12]. Dudchenko et al. [13] show that Hi-C data are less effective at correctly inferring fine scale structure, particularly when dealing with short contigs, and also show that orientation errors are generally more frequent than errors in chromosomal assignment or ordering of contigs within a chromosome, especially with short read data [13], supplemental table S3]. This is why it is recommended to scaffold from contigs with an N50 of at least 1 Mbp [21]. We also noted that correct orientation was less obvious in smaller contigs when reviewing Hi-C contact densities. This issue was especially pronounced in the repeat rich X chromosome (Fig. 3), and in the scaffolding of *M. monodon* which featured a lesser density of contacts overall.

Altogether, our results indicate that reducing the amounts of debris and inversion errors in reference genome construction will require methods that reduce the number of short contigs for scaffolding (Fig. 4). Long read datasets are a good start, but as shown here, not necessarily sufficient to eliminate these errors, a conclusion made most salient in the comparison of Mm_3_ to Mm_Damas_. Damas et al. [1] employed PacBio CLR and Dovetail Omni-C reads, the latter of which improve on Hi-C data by using sequence free endonucleases to achieve sequence independent, and thus even, contact coverage. Bionano data were also used (Damas pers. comm.), which uses optical genome mapping of sequence motifs to further scaffold the assembly. The comparisons made here suggest there are plateaus to assembly accuracy, and that the (nearly) final plateau is reached when employing methods used by Damas et al. [1] and research groups behind the Vertebrate Genomes Project ([6]; Fig. 4). Our results serve to remind end-users that reference genomes are an estimation, that the perception of quality is inflated without proper context, and that purging errors from assemblies requires several layers of datatypes that link large genomic distances.

The combination of methods used in assembly workflows also undoubtedly contributed to discrepancy across the reference genomes assessed. For instance, here we employed the long read assembler Flye [39], whereas Damas et al. [1] leveraged FALCON-unzip (Table 2). These assemblers are conceptually quite divergent; Flye emphasizes the resolution of repeat pathways while accounting for error rates, whereas FALCON emphasizes error correction by collapsing nested reads first, followed by assembly [40]. We can therefore expect differences in underlying principles to result in alternative assembly graphs and resulting sets of contigs, though the precise nature of these performative differences is beyond the scope of the current paper. Add to this differences in sequencing technology (e.g. PacBio vs Nanopore), downstream polishing or lack thereof, and scaffolding datatypes and procedures (e.g. Hi-C vs. Omni-C; 3D-dna vs instaGRAAL workflows [19]), and a beguiling array of workflow options become available, each step with the potential to impact the final assembly. Altogether, these differences in workflow construction simultaneously introduce variation in reference genome construction, while limiting our ability to pinpoint causation regarding assembly quality and putative errors.

Another important caveat to our results warrants consideration; we link the terms uncertainty and accuracy, and assume layered datatypes providing ultra-long distance genomic information converts uncertainty into accuracy. In other words, we assume reference genomes backed by methods such as those used by Damas et al. [1] are more accurate than its short read predecessors and even PacBio + HiC assembly. While it is reasonable to assume more and better data would naturally improve assembly accuracy, without knowing the true monodontid genomic sequences, this is impossible to validate. With this assumption in mind, however, our reference genome for beluga appears, in some respects, to be an improvement on the work of Jones et al. [22] and DNAzoo. There is clearly substantial room for improvement, however, following the example of Damas et al. [1] and the Vertebrates Genome Project [6]. We also note that the amounts of discrepancy in reference genomes reported here (Table 4) is likely to vary widely depending on the species, especially given the wide range in the amounts of repetitive elements and genome size across eukaryotic life (Fig. 4).

## Conclusions

### Considerations for reference genome construction in non-model organisms

Reference genomes are not infallible representations of species genomes. Oftentimes the context is lacking for understanding the nature of the uncertainties associated with reference genomes, or even the simple notion that these uncertainties exist. Consequently, the downstream impacts when leveraging a reference genome for analyses may not be fully understood. While poor to moderate genome quality nonetheless facilitates many biological questions, the limitation of assembly accuracy also precludes other evolutionary investigations (e.g. analyses of chromosomal evolution; intraspecific variation in genomic architecture; Fig. 4). In light of our analyses presented here, we forward several considerations for end-users seeking to leverage reference genomes in general. We hope these recommendations will be useful to neophytes constructing novel reference genomes of other species.

1. *Different project aims require different levels of scaffolding accuracy*: The importance of accuracy for a reference genome depends on study aims, and how those aims relate to positional information. For instance, low contiguity enables mapping reads for calling variant positions on a genome, while highly contiguous and accurately scaffolded assemblies enable the analysis of genomic regions or architecture (e.g., islands of differentiation). Highly accurate reference genomes are also needed to parse errors from naturally occurring variation, which can place a limitation on the study of genome structural evolution in closely related species or populations. End-users must therefore carefully scrutinize the data types and methods backing an assembly, and whether these support intended analyses.
2. *Accuracy should be prioritized over contiguity:* Chromosomal scale assemblies, in creating more junctions between contigs, may increase the potential number of mis-joins in a reference genome. End-users should therefore be mindful of disparity between the realized and true contiguity (i.e. inflated metrics), and that manual interventions are necessary to correct mis-joins (Fig. 1D). A conservative approach is favourable to over confidence introducing difficult to detect errors into reference genomes.
3. *Detecting regions of uncertainty requires multiple reference genomes*: The reference genomes presented here, had they been constructed in isolation, would have been considered perfectly valid. Mis-joins, scaffolding uncertainty, and errors in the ordering of contigs were revealed through cross comparisons with other reference genomes. While not benchmarking per se, replicate assemblies provide an additional layer of context to pin point regions of uncertainty in reference genomes, and assess whether a given reference genome(s) can be reliably applied to a biological question.
4. *Transparent methods and data sharing are essential*: Detailed methods, including command line arguments and accessions for publicly deposited read datasets, are critically important towards verifying the steps taken to arrive at a reference genome. Intermediate files should also be provided, including contig level assemblies and contact density information if Hi-C data were employed.

## Methods

### Generation of read data

Skin samples were obtained from three male belugas and one male narwhal harvested in the Hudson Bay-Strait Complex by Nunavik or Nunavut communities. Tissues were preserved in a saturated salt solution containing 20% dimethyl sulphoxide (DMSO) and 0.5 mol/L ethylene diamine tetraacetic acid (EDTA, [41]). DNA was extracted using the bead-based MagAttract HMW DNA Kit (Qiagen, Hilden, Germany) and quality was assessed using Pippin Pulse gel system (Sage Science, Beverly, Massachusetts). DNA extracts were sent to Genome Quebec for library preparation (SMTRbell Express Template Prep Kit 2.0, PacBio) and long reads sequencing on the Pacific BioSciences (PacBio) Sequel II system and for 150 bp paired-end short reads on the Illumina NovaSeq6000 S4.

Publicly available RNAseq data and Hi-C data for a female beluga and a male narwhal, respectively, were accessed through the Short Read Archive on July 4^th^, 2021 (Table S1). The Hi-C data for beluga were the same used for DNAzoo’s scaffolding of the Jones et al. [22] assembly (https://www.dnazoo.org/assemblies/Delphinapterus_leucas), and was generated using the restriction enzyme MboI (Olga pers. Comms). The Hi-C library for narwhal was generated using the restriction enzyme DpnII (Damas pers. Comms).

### Genome assembly and annotation

Subread files of long reads were converted to fasta using samtools v.1.13 [42], and read length distributions were assessed using the read lengths module in bbmap v.38.86 [43]. Reads were then subsampled to retain ca. 10 + kbp long reads for assembly (targeting ~ 50 × coverage), which were extracted using seqkit v.0.15.0 [44]. The subsampled long reads were assembled using Flye v.2.9 [39], which uses repeat graphs to reconstruct optimal assembly pathways.

Prior to polishing (i.e. correcting errors in long read assemblies using short reads), all short read datasets (paired-end, RNAseq, and Hi-C) were trimmed using trimmomatic v.0.39 [45] and evaluated using fastqc v.0.11.9 [46] and multiqc v.1.12 [47]. In general, trimming parameters were set to remove Illumina adapters, crop the first 10–15 bp, clip 3’ ends once quality values dipped below 20–25, retain reads with an average quality of 25, and retain reads with a minimum sequence length of 75 bp. Parameters were tailored for some datasets, in particular, some RNAseq datasets with shorter reads required an adjusted minimum length for read retention (set to 40 bp). Following this, the assembled genomes underwent two rounds of polishing ca. 75 × coverage using a combination of bowtie2 v.2.4.4 [48] and pilon v.1.24 [49].

Scaffolding was performed using the Juicer and 3D-dna workflows [12, 13] and publicly available Hi-C data (Table S1). Briefly, the Hi-C reads were mapped to the draft assembly using bwa [50]. Chimeric and duplicate reads were flagged and removed. Then, contigs were iteratively scaffolded and corrected for mis-join errors to achieve chromosomal-scale scaffolds based on contact densities, of which 22 were expected based on karyotype analysis [51]

The ordering and scaffolding of monodontid reference genomes were manually edited by visually inspecting contact densities using JuiceBox [37]. Specifically, scaffolding was edited by zooming in on the high-density diagonal line and manually correcting for mis-joins, inversions, and weakly supported scaffolding. We iteratively improved on the chromosomal scale assembly for this species by considering global scaffolding patterns across reference genomes (including that of DNAzoo for beluga) and triangulating large scale ordering. More precisely, if ordering was unequivocally supported in one reference genome, that information was carried over into the other reference genomes. Equally, if uncertainty was highlighted in one reference genome and revealed in others, breaks were introduced to the scaffolding. Finally, we performed a (second) final check for inversions for all our reference genomes by again carefully scrutinizing the high-density diagonal.

Genome stats for completeness and contiguity (total length, contig/scaffold max length, N50, L50) were calculated using the genome stats module in bbmap v.38.86 [43]. Genome completeness was evaluated using BUSCO v.5.2.2 [10], and was estimated using the vertebrata database (3,354 BUSCO markers). The X chromosome was identified by mapping our references to the *Bos taurus* X chromosome (CM008197.2) using minimap2 v.2.17 [52] using asm20 presets. The Y chromosome for beluga was not scaffolded given it was not present in the Hi-C data (see above).

Several lines of evidence were compiled for genome annotations. First, RNAseq datasets were assembled using rna-SPAdes v.3.15.4 ([53]; Table S1), and the standard transcripts output (not soft or hard filtered) were used as EST evidence. Vector contamination was removed from the assembled transcripts using SeqClean [54] and the UniVec core database [55]. To reduce computational demands downstream, the clean transcript datasets were pooled and mmseqs2 v.13–45111 [56] was used to cluster and extract representative sequences with settings tailored to prioritize longest transcripts, effectively removing duplicate transcripts from the datasets. Second, single copy universal genes identified through our BUSCO analysis for S_20_00703 (beluga) and S_20_00708 (narwhal) were pooled into single files, respectively, to be used as protein evidence for annotations in each species. Third, the repeat landscape was characterized de novo for each species using RepeatModeler v.2.0.3 [57] and with the -LTRStruct flag specified to further screen for Long Tandem Repeats (LTRs). The repeat libraries generated were then used as input to RepeatMasker v.4.1.2 [58], which was used to annotate repeat regions. Several dependencies were leveraged for the repeat analyses, including RMBlast v.2.11.0 [59], RECON v.1.08 [60], RepeatScout v.1.0.6 [61], Tandem Repeat Finder v.4.09.01 [62], LtrHarvest [63], Ltr_retriever [64], MAFFT v.7.505 [65], CD-HIT v.4.8.1 [66], and Ninja v.0.98-cluster_only [67]. To further reduce downstream computational demands, the repeat library was split into complex (LINEs, SINEs, and LTRs) and simple repeats (Satellites, short repeats, nucleotide specific rich areas) using grep commands. The complex repeats were stored as gff3 and converted into MAKER2 compatible formatting using perl v.5.30.2, while simple repeats were kept in fasta format.

The above evidence was used for an initial round of genome annotations using MAKER2 v.3.01.03 [68]. Gene models from round 1 of MAKER2 with a minimum length of 50 amino acids and AED scores of 0.25 or lower were then used to train ab initio gene models in SNAP (built 2017–05-17; [69]) and Augustus v.3.4.0 [70]. The Augustus model was additionally optimized using a built-in perl script. A second round of MAKER2 was performed, this time using the first round of annotations as input, thus avoiding costly realignments of transcriptomic, protein, and repeat evidence. The gene models for SNAP and Augustus were also specified. The MAKER2 round 2 results, as before, were then used to retrain SNAP and Augustus. The second round of ab initio gene models were used as input for a third round of MAKER2, as described above. The quality of the gene models was determined by examining the distribution in AED scores. MAKER2 built-in scripts were then used to extract protein sequences from the annotations. The protein sequences were blasted against the non-redundant reviewed Swiss-Prot database [71], and functional information was incorporated into the gene model annotations using MAKER2 built-in scripts.

All analyses were carried out on Compute Canada’s Cedar Cluster. The workflow is depicted in Figure S1, while the command lines with additional details are provided on GitHub: (https://github.com/tbringloe/Monodontid_assemblies_2023). Project files are available on FigShare (https://doi.org/10.6084/m9.figshare.23227595.v1), and sequence data was deposited with NCBI (BioProject PRJNA925093).

### Screening for discrepancies across reference genomes

Comparisons between new and previously published reference genomes were done using minimap2 v.2.17 [49] using asm5 presets. We used the most contiguous beluga reference genome, i.e., Dl_5_, for intraspecific comparisons with new and published reference genomes. We also compared our narwhal assembly to that of Damas et al. [1], but not to that of Westbury et al. [2] given this assembly was highly fragmented. Also, we assume the assembly of Damas et al. [1] is the most accurate of the analysed reference genomes, and is here regarded as a higher standard given the combined use of long reads, advanced types of chromosome capture data, and optical genome mapping. We therefore also assume the methods and data types of Jones et al. [22]; i.e. linked reads and iterative scaffolding) are the least accurate out of the approaches analysed.

Alignment blocks were filtered prior to screening for structural errors. Specifically, we removed alignment blocks with a query length < 3,000 bp, alignment lengths < 500 bp, mapQ < 30 (i.e. 1/1000 chance of a mapping error), and alignment match/length < 0.8. For the more fragmented genome of Jones et al. [22], we additionally filtered alignment blocks with a query length < 1 Mbp. In general, our approach mirrored those used by Quast v.5.0.2 [72], which is typically used to detect misassembly errors between genomes. Quast, however, characterizes misassemblies at the junction between alignment blocks, which did not facilitate quantifying discrepancy in bp (i.e., Quast calculates total misassembly length as the sum of all contigs with a misassembly detected).

We screened alignment files for four types of discrepancy, namely debris, translocations, inversions, and then relocations (Table 1). Note that we did not stack errors, such that unidentified inversions could be nested within translocations, and relocations could be nested within inverted alignment blocks. Moreover, in order to simplify our analysis, we did not consider unaligned blocks, discarded blocks (see above), or stacked alignment blocks (i.e. the shorter of query alignment blocks overlapping by > 50%) as discrepancies. Once alignment blocks were categorized, we summed the length of the query blocks assigned to each category and converted this to a percentage of total reference length. Discrepancies were also visualized as circos plots generated using CIRCA (http://omgenomics.com/circa). The relationship between total discrepancies and repetitive elements was also explored using regression analysis. Specifically, we compared discrepancy and repetitive elements as a percentage of chromosome lengths for each of the comparisons made, namely the PacBio beluga assemblies to each other, Jones et al. [22], and DNAzoo (Jones et al. [22] + HiC), and our PacBio narwhal assembly to Damas et al. [1].

### Supplementary Information

**Additional file 1: Table S1.** Specimen short read archive accession information for data used to generate improved genomes in beluga (Delphinapterus leucas) and narwhal (Monodon monoceros). **Figure S1.** Workflow diagram for the assembly and annotation of Delphinapterus leucas (Beluga) and Monodon monoceros (Narwhal) reference genomes

## Data Availability

Raw DNA sequence reads and genome assembly accessions may be found under SRA Bioproject PRJNA925093. Discrepancy tables, Hi-C contact matrices, assembly, annotation, and supporting files are available through FigShare (10.6084/m9.figshare.23227595.v1). Command line arguments and elaborations on the workflow can be accessed via github (https://github.com/tbringloe/Monodontid_assemblies_2023).

## Abbreviations

AED: Annotation Edit Distance
Dl: Delphinapterus leucas
Mm: Monodon monoceros
Mbp: Million base pairs
kbp: Thousand base pairs
