# Supplementary material for: Contrasting new and available reference genomes to highlight uncertainties in assemblies and areas for future improvement: an example with monodontid species
Source: BMC Genomics. 2023 Nov 20;24:693. doi: 10.1186/s12864-023-09779-3 (PMC10659057; doi:10.1186/s12864-023-09779-3)
Supplement: Supplementary file 1 — Additional file 1: Table S1. Specimen short read archive accession information for data used to generate improved genomes in beluga (Delphinapterus leucas) and narwhal (Monodon monoceros). Figure S1. Workflow diagram for the assembly and annotation of Delphinapterus leucas (Beluga) and Monodon monoceros (Narwhal) reference genomes [file 12864_2023_9779_MOESM1_ESM.docx]

### Table S1.

Specimen short read archive accession information for data used to generate improved genomes in beluga (*Delphinapterus leucas*) and narwhal (*Monodon monoceros*).

| **Species** | **Sample ID** | **Date collected** | **Location** | **Latitude** | **Longitude** | **PacBio long reads** | **Illumina short reads** | **RNA-seq** | **Hi-C** |
| --- | --- | --- | --- | --- | --- | --- | --- | --- | --- |
| *Delphinapterus leucas* | S_20_00693 | 2005-07 | Cumberland Sound; Pangnirtung | NA | NA | SRR23640103 | SRR23640100 |  |  |
| *Delphinapterus leucas* | S_20_00702 | 2002-07-22 | Cumberland Sound; Pangnirtung | NA | NA | SRR23640104 | SRR23640101 |  |  |
| *Delphinapterus leucas* | S_20_00703 | 2006-07-01 | Cumberland Sound; Pangnirtung | NA | NA | SRR23332917 | SRR23640098 |  |  |
| *Monodon monoceros* | S_20_00708 | 2001-09-01 | North Hudson Bay, Repulse Bay | NA | NA | SRR23640102 | SRR23640099 |  |  |
| *Monodon monoceros* | NGI_Narwhal_1 | 2013-08-08 |  | 72.3787 | 80.5310 |  |  | SRR8578702-5 | SRR8568868-70 |
| *Delphinapterus leucas* | Sample0237 | 2015-09-22 | SeaWorld | NA | NA |  |  |  | SRR8616923, SRR8616927 |
| *Delphinapterus leucas* | LC108772-12 | 2012-07-09 | Chuckchi Sea, Alaska | NA | NA |  |  | SRR6181300-3 |  |
| *Delphinapterus leucas* | DLBB-08-02 | 2008-05-17 | Bristol Bay, Alaska | NA | NA |  |  | SRR6181296-99 |  |
| *Delphinapterus leucas* | GAN/ISIS: 26980492/103006 (dam) | NA | Vancouver Aquarium | NA | NA |  |  | SRR5282288, SRR5282291-2, SRR5282294-8, SRR5990716-8 |  |
| *Delphinapterus leucas* | GAN/ISIS: 26980489/103008 | NA | Vancouver Aquarium | NA | NA |  |  | SRR5282283-7, SRR5282289-90, SRR5282293 |  |
| *Delphinapterus leucas* | DLBB12-01 | 2012-09-01 | Bristol Bay, Alaska | 59.034033 | -158.3954 |  |  | SRR5521314 |  |
| *Delphinapterus leucas* | DLBB12-02 | 2012-09-01 | Bristol Bay, Alaska | 59.053067 | -158.391983 |  |  | SRR5521315 |  |
| *Delphinapterus leucas* | DLBB12-03 | 2012-09-08 | Bristol Bay, Alaska | 59.053067 | -158.391983 |  |  | SRR5521316 |  |
| *Delphinapterus leucas* | DLBB12-04 | 2012-09-08 | Bristol Bay, Alaska | 59.055417 | -158.4083 |  |  | SRR5521317 |  |
| *Delphinapterus leucas* | DLBB12-05 | 2012-09-09 | Bristol Bay, Alaska | 58.590467 | -158.501517 |  |  | SRR5521318 |  |
| *Delphinapterus leucas* | DLBB12-06 | 2012-09-10 | Bristol Bay, Alaska | 58.590467 | -158.501517 |  |  | SRR5521319 |  |
| *Delphinapterus leucas* | DLBB12-07 | 2012-09-12 | Bristol Bay, Alaska | 58.8623 | -158.705267 |  |  | SRR5521320 |  |
| *Delphinapterus leucas* | DLBB12-09 | 2012-09-12 | Bristol Bay, Alaska | 58.7598 | -158.7742 |  |  | SRR5521321 |  |
| *Delphinapterus leucas* | DLBB13-01 | 2013-08-23 | Bristol Bay, Alaska | 59.0263 | -158.4287 |  |  | SRR5521322 |  |
| *Delphinapterus leucas* | DLBB13-02 | 2013-08-24 | Bristol Bay, Alaska | 59.0530 | -158.3958 |  |  | SRR5521323 |  |
| *Delphinapterus leucas* | DLBB13-03 | 2013-08-24 | Bristol Bay, Alaska | 59.05109 | -158.38335 |  |  | SRR5521324 |  |
| *Delphinapterus leucas* | DLBB13-04 | 2013-08-24 | Bristol Bay, Alaska | 59.0329 | -158.36238 |  |  | SRR5521325 |  |
| *Delphinapterus leucas* | DLBB13-07 | 2013-08-28 | Bristol Bay, Alaska | 59.0133 | -158.4614 |  |  | SRR5521326 |  |
| *Delphinapterus leucas* | DLBB13-08 | 2013-08-28 | Bristol Bay, Alaska | 59.0539 | -158.3981 |  |  | SRR5521327 |  |
| *Delphinapterus leucas* | DLBB13-09 | 2013-08-30 | Bristol Bay, Alaska | 59.0190 | -158.4417 |  |  | SRR5521328 |  |
| *Delphinapterus leucas* | DLBB13-10 | 2013-08-30 | Bristol Bay, Alaska | 58.8934 | -158.5139 |  |  | SRR5521329 |  |
| *Delphinapterus leucas* | DLBB14-01 | 2014-08-25 | Bristol Bay, Alaska | 59.05273 | -158.3881 |  |  | SRR5521330 |  |
| *Delphinapterus leucas* | DLBB14-03 | 2014-08-26 | Bristol Bay, Alaska | NA | NA |  |  | SRR5521331 |  |
| *Delphinapterus leucas* | DLBB14-05 | 2014-08-28 | Bristol Bay, Alaska | 59.0517 | -158.3657 |  |  | SRR5521332 |  |
| *Delphinapterus leucas* | DLBB14-06 | 2014-08-28 | Bristol Bay, Alaska | 58.95997 | -158.49783 |  |  | SRR5521333 |  |
| *Delphinapterus leucas* | DLBB14-07 | 2014-08-29 | Bristol Bay, Alaska | NA | NA |  |  | SRR5521334 |  |
| *Delphinapterus leucas* | DLBB14-08 | 2014-08-31 | Bristol Bay, Alaska | 58.855289 | -158.677329 |  |  | SRR5521335 |  |
| *Delphinapterus leucas* | DLBB14-09 | 2014-08-31 | Bristol Bay, Alaska | 58.816445 | -158.674507 |  |  | SRR5521336 |  |
| *Delphinapterus leucas* | DLBB14-10 | 2014-09-03 | Bristol Bay, Alaska | 58.816445 | -158.674507 |  |  | SRR5521337 |  |

**
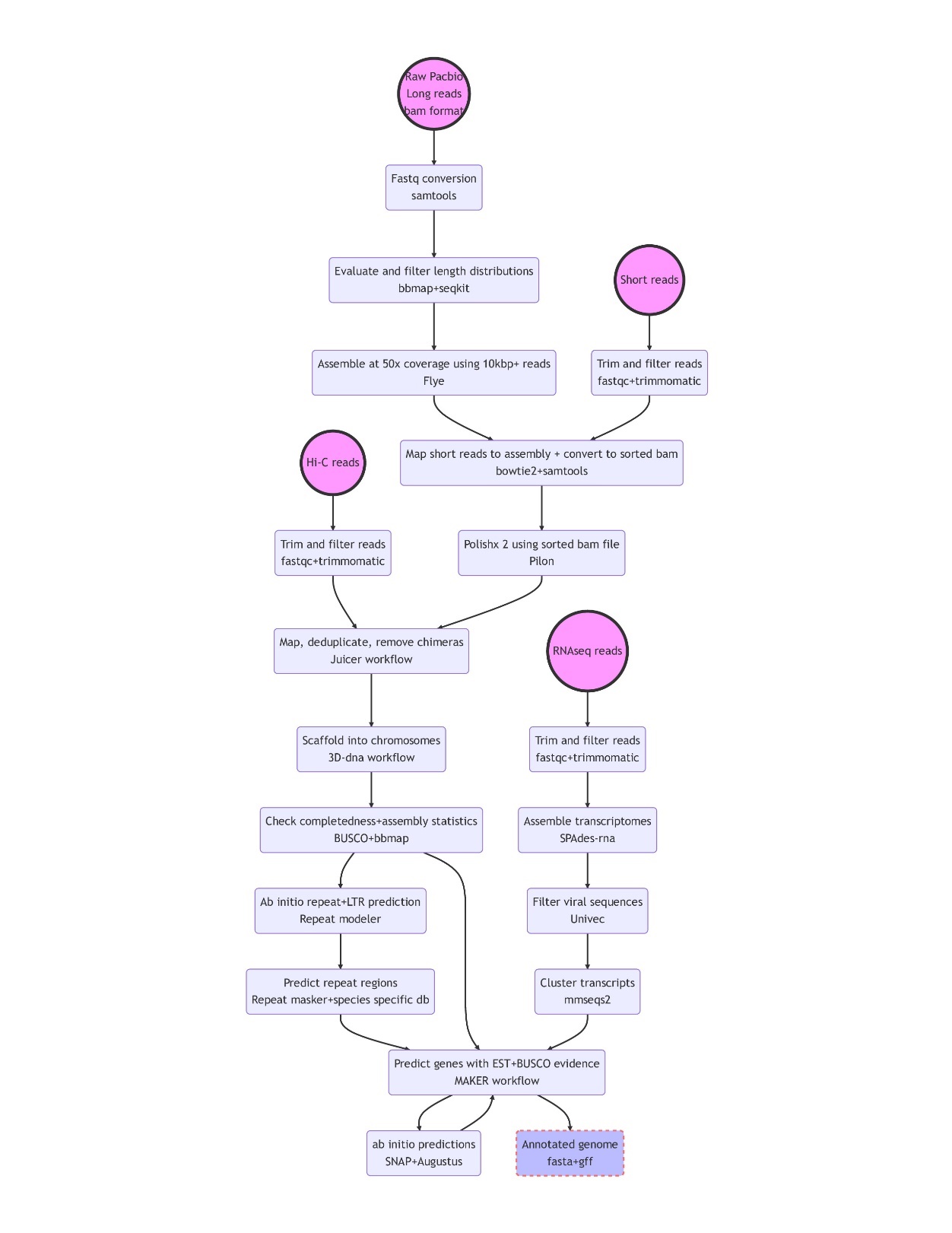
**

### Figure S1.

Workflow diagram for the assembly and annotation of *Delphinapterus leucas* (Beluga) and *Monodon monoceros* (Narwhal) reference genomes.
